# Sodium Hypochlorite 0.005% Versus Placebo in the Treatment of Mild to Moderate Acne: A Double-Blind Randomized Controlled Trial

**DOI:** 10.5826/dpc.1103a46

**Published:** 2021-05-20

**Authors:** Azadeh Dorostkar, Mehdi Ghahartars, Mohammadreza Namazi, Nafiseh Todarbary, Maryam Hadibarhaghtalab, Maryam Rezaee

**Affiliations:** 1 Dermatology Department, Shahid Faghihi Hospital, Shiraz University of Medical Sciences, Shiraz, Iran; 2 Student Research Committee, Shiraz University of Medical Sciences, Shiraz, Iran

**Keywords:** topical, sodium hypochlorite, acne vulgaris, treatment

## Abstract

**Background:**

Acne vulgaris is a common inflammatory disease of the pilosebaceous follicle that affects many teenagers and young people. There is an obvious need for topical treatments with good tolerability and efficacy for the management of acne lesions.

**Objective:**

This study determined the therapeutic efficacy of topical sodium hypochlorite solution (0.005%) in the treatment of mild to moderate acne lesions.

**Methods:**

This placebo-controlled randomized controlled trial compared 0.005% sodium hypochlorite to placebo administered topically on each side of the patients’ faces 3 times a day for 1 month. The numbers of papules and pustules were recorded at baseline, 1, 2 and 4 weeks after initiation.

**Results:**

The total number of papules and pustules decreased after topical application of sodium hypochlorite 0.005% for 1 month.

**Conclusions:**

Topical sodium hypochlorite solution (0.005%) can be effective in the treatment of mild to moderate acne, and its clinical efficacy was evaluated between the male and female groups and between the hormonal and non-hormonal ones.

**Trial registration:**

Our study was registered in the Iranian Registry of Clinical Trials (IRCT) with the code number IRCT20200701047976N1.

## Introduction

Acne vulgaris, the most common dermatological disease, affects 35%–90% of adolescents [1]. Acne is inflammation of the pilosebaceous units of the skin, and it presents as lesions such as scars and hyperpigmentation [2]. Although the ideal treatment for acne is still under investigation, an effective regimen to reduce lesions can be found for most patients. However, there is a lack of high-quality evidence on the comparative effectiveness of common topical and systemic acne treatments [3]. Additionally, the complex combination treatment regimens that affect different aspects of acne pathophysiology can lead to poor treatment adherence and may decrease the effectiveness of the treatment [4].

Sodium hypochlorite is a common antiseptic agent with a broad application in medicine, including use in bladder and urethra irrigation, control of some topical mycoses, prophylaxis against burn infections, and as an irrigant in dental root canal procedures [5]. In 2017, Eriksson and colleagues revealed that sodium hypochlorite 0.004% considerably decreased the *S. aureus* load in atopic dermatitis lesions [6]. Additionally, Hunter revealed that sodium hypochlorite accelerated the remission of herpes simplex lesions [5]. We hypothesized that sodium hypochlorite 0.005% is possibly effective in treating mild to moderate acne. Therefore, this placebo-controlled, randomized controlled trial investigated the therapeutic efficacy of sodium hypochlorite 0.005% in the treatment of mild to moderate acne.

## Methods

This randomized controlled trial was conducted on 40 consecutive acne patients seen at the Dermatology Clinic of Shahid Faghihi Hospital, from November 2017 to April 2018. Patients with secondary acne caused by lactation, dermatology conditions, or allergy to sodium hypochlorite as well as those who had taken isotretinoin or systemic or topical antibiotics within the past 2 months were excluded from the study. The review board of Shiraz University of Medical Sciences approved the study, and all recruited patients filled the informed consent form. The study was registered in the Iranian Registry of Clinical Trials (IRCT) with the code number IRCT20200701047976N1.

The patients were randomly assigned to receive sodium hypochlorite 0.005% or placebo, to be administered topically on each side of their face 3 times a day for 1 month. The patients were not aware of the type of solution (placebo or sodium hypochlorite) applied to their faces. Moreover, the dermatologist and the observer were blinded to the identity of the solution applied to each patient.

We used the modified global acne grading scale (mGAGS) to assess the severity of acne during the study.

Statistical Package for Social Science for Windows was used for data analysis. Because the data were non-parametric, we used the Wilcoxon test to compare the numbers of lesions between the two groups. Additionally, we used the Friedman test to evaluate changes in the number of papules and pustules during the study. A P-value <0.05 was considered statistically significant.

## Results

The study enrolled 40 patients with mild to moderate acne, including 32 (80%) women and 8 (20%) men. The mean age of the patients was 21.3 years (range, 15–28 years). After one month, the total number of papules and pustules decreased significantly, from 759 to 476, in the patients (P < 0.0001). Moreover, the number of papules decreased significantly after one month (P < 0.0001), while the number of pustules did not change significantly (P = 0.692). Table 1 shows the statistical analysis of papules and pustules over 1 month.

Table 2 shows the statistical analysis in hormonal and non-hormonal groups. The number of papules and pustules did not change significantly in these groups over 1 month (in both groups; P > 0.05).

The number of pustules decreased in the female group (P = 0.005) but the number of papules did not change remarkably (P >0.05). Table 3 shows the statistical analysis for males vs females. Topical application of sodium hypochlorite caused more acne remission in the female group than in the male group (P > 0.05).

## Discussion

Our study shows that the total number of acne lesions decreased significantly after applying sodium hypochlorite 0.005% topically 3 times a day for 1 month. Consequently, topical application of sodium hypochlorite 0.005% can be effective in the treatment of mild to moderate acne.

A reduction in the total number of papules and pustules highlights the antimicrobial properties of sodium hypochlorite. Del Rosso and colleagues reported that sodium hypochlorite was an effective antimicrobial agent to decrease the load of *S. aureus* in atopic dermatitis lesions [8]. Coetzee and colleagues indicated that an un-buffered solution of sodium hypochlorite 0.006% was effective in the management of infected burn wounds caused by *Pseudomonas aeruginosa*, *S. aureus* and *Streptococcus pyogenes* [9]. Consequently, sodium hypochlorite can possibly be helpful to decrease the load of *Propionibacterium acnes* in acne lesions.

A reduction of pustules in the female group was associated with the topical application of sodium hypochlorite 0.005%. Consequently, we can use it as a treatment for female acne similar to other topical products [10]. Preneau and Dreno showed that female acne is a common female disease (40%–50%) that should be treated differently from adolescent acne because of differences in the clinical scale and therapeutic algorithm [11].

Acne therapy comprises several options, but the efficacy and adverse events of each treatment are under investigation. By way of illustration, treatment with oral antibiotics is long-lasting because of bacterial resistance, adverse effects and low efficacy during short-term application. Additionally, acne therapy with isotretinoin is teratogenic with noticeable side effects; thus, it needs close follow-up. Hormonal therapy is limited to female patients and its side effects include abnormal menstruation, nausea and vomiting, weight gain, breast tenderness, and higher risk of thromboembolism [12]. Furthermore, costs and availability are considerable factors of treatment, especially in developing countries. Therefore, sodium hypochlorite 0.005% can be a suitable option for acne treatment due to its low cost, high availability and few side effects.

Sodium hypochlorite 0.005% was safe and well-tolerated by patients of our study, but skin-related adverse events such as skin irritation or itching, and acne reoccurrence have been our concerns after discontinuation of sodium hypochlorite 0.005%.

Studies have revealed that combination therapy is more successful in acne treatment. As a result, the combination of sodium hypochlorite 0.005% with retinoids or antibiotics can improve the anti-inflammatory effects of the treatment regimen or reduce the resistance to antibiotics, respectively [2]. Additionally, the combination with compounds such as azelaic acid, salicylic acid and tazarotene can be effective in acne therapy. As Hidalgo et al.’s study showed, the bactericidal effect of sodium hypochlorite increases by reducing the pH [5].

Because our study group was small, more studies with more patients are needed to determine the clinical efficacy of sodium hypochlorite 0.005% in acne therapy.

In conclusion, our randomized controlled trial demonstrates that sodium hypochlorite 0.005% is an effective and safe treatment for acne, and that the therapeutic outcomes were different between hormonal and non-hormonal acne and between male and female groups.

## Figures and Tables

**Table 1:** Mean rank of papules and pustules in 40 acne patients who received either hypochlorite sodium or placebo over one month

		Hypochlorite group	Placebo group	P value
**Papules**	**Week 1**	9.83	9.33	0.319
	**Week 2**	6.99	7.78	0.266
	**Week 4**	5.85	7.66	0.015
**Pustules**	**Week 1**	7.99	5.28	0.003
	**Week 2**	4.14	5.25	0.029
	**Week 4**	2.93	5.00	0.002

**Table 2:** Ranking of papules and pustules in the patients with hormonal and non-hormonal acne who received hypochlorite sodium or placebo over 1 month.

		Hypochlorite group	Placebo group	P value

**Hormonal papules**	**Week 1**	3.00	7.00	0.673

	**Week 2**	5.33	5.75	0.645

	**Week 4**	5.75	5.13	0.473

**Hormonal pustules**	**Week 1**	3.67	5.67	0.170
	
	**Week 2**	5.81	4.25	0.051
	
	**Week 4**	5.13	4.00	0.028

**Non-hormonal papules**	**Week 1**	10.15	16.08	0.410

	**Week 2**	11.47	13.00	0.299

	**Week 4**	14.53	12.50	0.014

**Non-hormonal pustules**	**Week 1**	8.75	13.15	0.009

	**Week 2**	14.88	9.68	0.206

	**Week 4**	13.53	11.64	0.028

**Table 3:** Mean rank of papules and pustules in the male and female patients who received hypochlorite sodium or placebo over 1 month.

		Hypochlorite group	Placebo group	P value
**Male papules**	**Week 1**	4.14	7.00	0.107
	**Week 2**	4.92	5.17	0.399
	**Week 4**	5.21	4.25	0.095
**Male pustules**	**Week 1**	3.25	4.30	0.201
	**Week 2**	5.33	4.33	0.252
	**Week 4**	4.67	4.00	0.158
**Female papules**	**Week 1**	9.59	15.68	0.124
	**Week 2**	11.50	14.17	0.518
	**Week 4**	14.97	13.50	0.062
**Female pustules**	**Week 1**	9.21	14.47	0.008
	**Week 2**	15.47	9.30	0.058
	**Week 4**	14.25	11.00	0.005
